# Cardiotoxicity of 5-fluorouracil and capecitabine in Chinese patients: a prospective study

**DOI:** 10.1186/s40880-018-0292-1

**Published:** 2018-05-11

**Authors:** Jianjun Peng, Chao Dong, Chang Wang, Weihua Li, Hao Yu, Min Zhang, Qun Zhao, Bo Zhu, Jun Zhang, Wenliang Li, Fenghua Wang, Qiong Wu, Wenhao Zhou, Ying Yuan, Meng Qiu, Gong Chen

**Affiliations:** 1 Center of Gastrointestinal Surgery, First Affiliated Hospital of Sun Yat-sen University, Guangzhou, 510080 Guangdong P. R. China; 2 Department of the Second Medical Oncology, The 3rd Affiliated Hospital of Kunming Medical University, Yunnan Tumor Hospital, Kunming, 650118 Yunnan P. R. China; 3 Department of Oncology, The First Hospital of Jilin University, Changchun, 130021 Jilin P. R. China; 4 Department of Surgical Oncology, Fujian Provincial Hospital, Fuzhou, 350014 Fujian P. R. China; 5 Department of Biological Statistics, School of Public Health, Nanjing Medical University, Nanjing, 211166 Jiangsu P. R. China; 6 Department of Cardiology, Shanghai Jiaotong University, Shanghai, 200030 P. R. China; 7 The Third Surgery Department, the Fourth Hospital of Hebei Medical University, Tumor Hospital of Hebei Province, Shijiazhuang, 050011 Hebei P. R. China; 8 Institute of Cancer, Xinqiao Hospital of Third Military Medical University, Chongqing, 400037 P. R. China; 9 Oncology Department, Shanghai Ruijin Hospital, Shanghai, 200025 P. R. China; 10 Oncology Department, First Affiliated Hospital of Kunming Medical University, Kunming, 650032 Yunnan P. R. China; 11 Department of Internal Medicine, Sun Yat-sen University Cancer Center, Guangzhou, 510060 Guangdong P. R. China; 12 Medical Oncology, The first Affiliated Hospital of Bengbu Medical College, Bengbu, 233004 Anhui P. R. China; 13 Department of Colorectal Surgery, Sun Yat-sen University Cancer Center, 651 Donfeng Rd, East, Guangzhou, 510060 Guangdong P. R. China; 14 Medical Oncology, The Second Affiliated Hospital of Zhejiang University School of Medicine, Hangzhou, 310009 Zhejiang P. R. China; 15 Department of Medical Oncology, Cancer Center, The State Key Laboratory of Biotherapy, West China Hospital, West China Medical School, Sichuan University, No. 37, Guo Xue Xiang, Chengdu, 610041 Sichuan P. R. China

**Keywords:** 5-Fluorouracil, Capecitabine, Cardiotoxicity

## Abstract

**Background:**

5-Fluorouracil (5-FU) and capecitabine-associated cardiotoxicity ranging from asymptomatic electrocardiography (ECG) abnormalities to severe myocardial infarction has been reported in a number of studies, but such cardiotoxicity in Chinese patients with malignant diseases has not been investigated to date. In the present study, we aimed to prospectively evaluate the incidence rates and clinical manifestations of 5-FU- and capecitabine-associated cardiotoxicity in cancer patients recruited from multiple centers in China.

**Methods:**

Among the 527 patients who completed the study, 196 received 5-FU-based chemotherapy and 331 received capecitabine-based chemotherapy as either first-line or adjuvant therapy. Adverse events were reported during the treatment and up to 28 days of follow-up. Outcome measures included ECG, myocardial enzymes, cardiac troponin, brain natriuretic peptide and echocardiography. Univariate analysis and logistic regression were performed for subgroup analysis and identification of significant independent variables that are associated with cardiotoxicity of both agents.

**Results:**

In total, 161 of 527 patients (30.6%) experienced cardiotoxicity. The incidence rate of cardiotoxicity was 33.8% (112/331) in the capecitabine group, which was significantly higher than the rate of 25% (49/196) in the 5-FU group (*P* = 0.0042). 110/527 patients (20.9%) suffered arrhythmia, 105/527 (19.9%) developed ischemic changes, while only 20/527 patients (3.8%) presented heart failure and 6/527 patients (1.1%) had myocardial infarction. Pre-existing cardiac disease, hypertension, capecitabine-based chemotherapy and duration of treatment were identified as significant risk factors associated with cardiotoxicity. The odds ratio were 15.7 (prior history of cardiac disease versus no history), 1.86 (capecitabine versus 5-FU), 1.06 (5–8 versus 1–4 chemotherapy cycles) and 1.58 (hypertension versus no hypertension), respectively.

**Conclusions:**

Cardiotoxicity induced by fluoropyrimidines in the Chinese population may be underestimated in clinical practice. Close monitoring of patients is recommended, especially for those patients at high risk for cardiotoxicity. Possible risk factors are duration of treatment, capecitabine-based chemotherapy, pre-existing cardiac diseases and hypertension.

*Trial registration* This study was initiated on January 22, 2014 and has been retrospectively registered with the registration number ChiCTR1800015434

## Background

Over the past decades, there has been an increasing number of patients presenting oncologic and cardiologic co-morbidities [1]. A substantial proportion of patients who underwent chemotherapy showed different degrees of cardiac dysfunction. However, prevention of such cardiac morbidities through early diagnosis is still an unmet need. Recently, cardio-oncology has emerged as a new discipline to provide expertise in the development of innovative strategies and interdisciplinary therapies for cardiologic and oncologic co-morbidities [1]. The chemotherapeutic drug 5-fluorouracil (5-FU) and its oral prodrug capecitabine have been reported to induce adverse cardiac effects, despite being considered as tolerable in most cases [2–4]. 5-FU and capecitabine are pyrimidine antimetabolites that have been widely used in the treatment of various malignant diseases. Emerging studies have shown that they can induce a spectrum of adverse cardiac effects including chest pain, dyspnea and hypotension [5–7]. Other clinical presentations include arrhythmias, heart failure, myocardial infarction, cardiogenic shock and sudden death [4–9]. A recent meta-analysis indicates that the incidence of symptomatic cardiotoxicity is 0–20% in patients treated with 5-FU and 3–35% in patients receiving capecitabine [10]. While 5-FU- and capecitabine-associated cardiotoxicity has been widely addressed in the US and European countries, the incidence and risk factors for such cardiotoxicity, to our knowledge, has never been prospectively investigated in Chinese patients. In this study, we aimed to prospectively evaluate the incidence, clinical manifestations and risk factors of 5-FU- and capecitabine-associated cardiotoxicity in patients recruited from multiple centers in China.

## Patients and methods

### Patients

Patients were consecutively recruited from 12 medical centers in China between January 2014 and March 2016. Inclusion criteria for the study included: age ≥ 18 years old; histologically, pathologically or clinically diagnosed solid tumors including colorectal, gastric, esophageal, breast, and head and neck carcinomas; patients receiving 5-FU or oral capecitabine-based chemotherapy regimens. A history of cardiac diseases was not an exclusion criterion for the study. Patients who required proactive termination or change of the chemotherapeutic regimen were excluded from analysis. All patients underwent a physical examination, electrocardiography (ECG), echocardiography, chest X-ray and blood tests including blood cell count and serum biochemical analysis, prior to assignment to study treatment with 5-FU or capecitabine. Written informed consents were obtained from all patients before their participation. The study was approved by the institutional review boards of all participating centers.

### 5-FU and capecitabine treatment

Patients were assigned to either 5-FU or capecitabine treatment based on their disease status, economic condition and their own intention. 5-FU-based chemotherapy was given according to the mFOLFOX6 regimen. Specifically, the doses were given in 2-week cycles. Oxaliplatin and leucovorin were given via i.v. infusion at doses of 85 and 400 mg/m^2^ respectively on day 1, followed by administration of 5-FU as i.v. bolus at a dose of 400 mg/m^2^ on day 1, then 2400 mg/m^2^ over 46–48 h. Capecitabine-based chemotherapy was given in 21-day cycles as a 2-week-on/1-week-off regimen. For patients receiving capecitabine monotherapy, the dose was 1250 mg/m^2^, twice daily from day 1 to day 14 followed by a 7-day rest period. For patients receiving capecitabine in combination with oxaliplatin, oxaliplatin was given as a 2-h i.v. infusion at a dose of 130 mg/m^2^ on day 1 and oral capecitabine given at 1000 mg/m^2^ twice daily from day 1 to day 14.

### Evaluation of cardiotoxicity

All patients included in the study were examined by ECG before and after each chemotherapy cycle. During the chemotherapy treatment with 5-FU or capecitabine, patients who complained of cardiotoxicity were evaluated by a cardiologist based on the clinical symptoms and laboratory tests including ECG, myocardial enzymes, cardiac troponin, brain natriuretic peptide and echocardiography. In patients who presented with evidence of cardiotoxicity, the following work-up was adopted based on the condition of each patient: initiation of cardiac monitoring, dose reduction of 5-FU or capecitabine, use of sublingual nitrates or calcium antagonists, or change to other chemotherapeutic drugs. Patients were followed up until cardiotoxicity disappeared or became stably controlled. Patients who did not present cardiotoxicity throughout the duration of chemotherapy would be subsequently followed up for 4 weeks.

### Statistical analysis

Homogeneity in the distribution of frequency data between groups was examined using Pearson’s χ^2^ test or Fisher’s exact test. The effects of potential risk factors including sex, age, pre-existing cardiac disease, hypertension, chemotherapeutic agent and chemotherapy cycles on cardiotoxicity were determined using stepwise logistic regression. A *P* value < 0.05 was considered statistically significant. All statistical analyses were performed using the SPSS software, version 19.0 (IBM, Armonk, NY, USA).

## Results

### Patient characteristics

A total of 532 patients were consecutively recruited from 12 medical centers in China between January 2014 and March 2016. Patients who required proactive termination or change of the chemotherapeutic regimens were excluded from the analysis (n = 5). A total of 527 patients were included in the final analysis, including 348 men and 179 women with a mean age of 57 (ranged 23–87). The majority of patients had primary colorectal (71.7%) and gastric cancers (23.3%). Nearly half of the cancers were in stage III and 29.0% in stage IV. The patient characteristics at baseline are listed in Table 1.

**Table 1:** Primary cancer characteristics and pre-existing disease of patients at baseline

Variable	Patients [cases (%)]
Total	527
Male	348 (66.0)
Primary cancer	
Colorectal	378 (71.7)
Gastric	123 (23.3)
Head and neck	15 (2.9)
Esophageal	6 (1.1)
Breast	4 (0.8)
Nasopharyngeal	1 (0.2)
Cancer stage	
I	2 (0.4)
II	105 (20.0)
III	249 (47.2)
IV	153 (29.0)
Not available	18 (3.4)
Pre-existing disease	
Hypertension	106 (20.1)
Diabetes	29 (5.5)
Cardiac disease	13 (2.5)
Hyperlipidemia	4 (0.8)
Surgical history	415 (78.7)
Radical	338 (64.1)
Palliative	57 (10.8)
Unknown	20 (3.8)
Treatment history	176 (33.4)
Chemotherapy	118 (22.4)
Radiotherapy	32 (6.1)
Chemotherapy and radiotherapy	26 (4.9)

### Treatment protocol

The treatment plan was based on the standard protocol (Table 2). A total of 196 patients (37.2%) received 5-FU-based treatment and 331 (62.8%) received capecitabine-based treatment. Within the 5-FU treatment group, the majority of patients (93.9%) were treated with a combination of other chemotherapeutic agents. In the capecitabine group, 11 patients (3.3%) received capecitabine as monotherapy and the remaining 320 (96.7%) received capecitabine in combination with other anticancer drugs.

**Table 2:** Proportion of patients receiving different 5-FU- and capecitabine-based chemotherapeutic regimens

Treatment protocol	Patients	
	n	%
5-FU based treatment regimen	196	
5-FU bolus	3	1.5
24-h continuous infusion	9	4.6
5-FU + folinic acid + oxaliplatin	118	60.2
5-FU + folinic acid + irinotecan	37	18.9
Other 5-FU combo therapy	29	14.8
Capecitabine based treatment regimen	331	
Capecitabine monotherapy	11	3.3
Capecitabine + oxaliplatin	310	93.7
Other capecitabine combo therapy	10	3.0

5-FU: 5-Fluorouracil

Around 50% of the patients completed five to eight chemotherapy, whereas around 37% of the patients had less than four chemotherapy cycles (Table 3).

**Table 3:** The number of chemotherapy cycles completed by the patients

Treatment cycle	All patients, n (%) (*n *= 527)	5-fluorouracil, n (%) (*n *= 196)	Capecitabine, n (%) (*n *= 331)
1–4	196 (37.2)	68 (34.7)	128 (38.7)
5–8	268 (50.9)	91 (46.4)	177 (53.5)
≥ 9	35 (6.6)	32 (16.3)	3 (0.9)
Unknown	28 (5.3)	5 (2.6)	23 (6.9)

### Cardiotoxicity in patients who underwent 5-FU or capecitabine chemotherapy

We found that 161 patients (30.6%) in the study developed clinical symptoms of cardiotoxicity, with 49 (25.0%) in the 5-FU group and 112 (33.8%) in the capecitabine group. The main clinical manifestations were arrhythmia (20.9%) and ischemic change (19.9%). Twenty patients (3.8%) developed heart failure, 6 patients (1.1%) had myocardial infarction and 21 patients (4.0%) reported chest discomfort and palpitation (Table 4). The majority of patients had their first cardiac complication during the first (42.2%) and the second (24.8%) chemotherapy cycles. When we excluded the 13 patients who had a history of cardiac disease (eight with arrhythmia, two with coronary heart disease, one with valvular disease, one with hypertensive heart disease and one with myocardial ischemia), the incidence rates of symptomatic cardiotoxicity remained the same (29.4% vs 30.6%). Specifically, 103 patients (20.0%) developed arrhythmia, 98 (19.1%) had ischemic change, 19 (3.7%) had heart failure, 6 (1.2%) developed myocardial infarction and 21 (4.1%) presented chest discomfort.

**Table 4:** Incidence of various clinical manifestations of cardiotoxicity in the patients

Clinical manifestations	All patients (*n *= 527)	5-fluorouracil (*n *= 196)	Capecitabine (*n *= 331)
Heart failure	20 (3.8)	5 (2.6)	15 (4.5)
Myocardial infarction	6 (1.1)	2 (1.0)	4 (1.2)
Arrhythmia	110 (20.9)	33 (16.8)	77 (23.3)
Sinus bradycardia	42 (8.0)	11 (5.6)	31 (9.1)
Premature beat	33 (6.3)	10 (5.1)	23 (7.0)
Conduction block	28 (5.3)	9 (4.6)	19 (5.7)
Sinus tachycardia	26 (4.9)	11 (5.6)	15 (4.5)
Atrial fibrillation	2 (0.4)	0 (0.0)	2 (0.6)
Ischemic change	105 (19.9)	39 (19.9)	66 (19.9)
ST segment	70 (13.3)	23 (11.7)	47 (14.2)
T segment	48 (9.1)	21 (10.7)	27 (8.2)
P segment	9 (1.7)	3 (1.5)	6 (1.8)
R segment	3 (0.6)	1 (0.5)	2 (0.6)
Total patients	161 (30.6)	49 (25.0)	112 (33.8)

Data are represented as n (%)

### Factors associated with cardiotoxicity

Potential risk factors were analyzed for their individual association with cardiotoxicity in the cohort. Pre-existing cardiac disease, hypertension and capecitabine-based chemotherapeutic regimen significantly increased the risk of developing cardiotoxicity (all *P* < 0.05; Table 5). By using stepwise logistic regression, we found that patients with pre-existing cardiac diseases had a 15.7-fold increased risk of developing cardiotoxicity compared with those without and capecitabine based chemotherapy was more likely to induce cardiotoxicity compared to 5-FU treatment. Prior history of cardiac disease versus no history: OR 15.7, 95% CI 1.76–139.76, *P* = 0.01; capecitabine versus 5-FU treatment: OR 1.86, 95% CI 1.13–3.04, *P* = 0.01; prior history of hypertension versus no history: OR 1.58, 95% CI 0.90–2.77, *P* = 0.11; chemotherapy cycles: OR 1.06 (increased by each cycle), 95% CI 1.00–1.15, *P* = 0.21; age: OR 1.00 (age range is 23–87), 95% CI 0.82–1.22, *P* = 1.00; male versus female: OR 0.87, 95% CI 0.54–1.40, *P* = 0.57.

**Table 5:** Risk factors tested for association with 5-FU- and capecitabine-associated cardiotoxicity

Variable	Patients with cardiotoxicity (*n *= 161)	Patients without cardiotoxicity (*n *= 366)	*P*
Chemotherapy			
5-Fluorouracil	49 (30.4)	147 (40.2)	0.033
Capecitabine	112 (69.6)	219 (59.8)	
Chemotherapy cycle			
1–4	53 (32.9)	143 (39.1)	0.386
5–9	91 (56.5)	177 (48.4)	
≥ 9	9 (5.6)	26 (7.1)	
UK	8 (5.0)	20 (5.5)	
Concurrent radiotherapy			
Yes	6 (3.7)	16 (4.4)	0.733
No	155 (96.3)	350 (95.6)	
Sex			
Male	108 (67.1)	240 65.6)	0.737
Female	53 (32.9)	126 (34.4)	
Age, years			
≤ 29	4 (2.5)	6 (1.6)	0.257
30~	9 (5.6)	23 (6.3)	
40~	33 (20.5)	79 (21.6)	
50~	40 (24.8)	110 (30.1)	
60~	54 (33.5)	124 (33.9)	
70~	19 (11.8)	23 (6.3)	
80~	2 (1.2)	1 (0.3)	
Pre-existing disease			
Hypertension			
Yes	44 (27.3)	62 (16.9)	0.006
No	117 (72.7)	304 83.1)	
Diabetes			
Yes	11 (6.8)	18 (4.9)	0.375
No	150 (93.2)	348 (95.1)	
Cardiac disease			
Yes	9 (5.6)	4 (1.1)	0.004
No	152 (94.4)	362 (98.9)	
Hyperlipidemia			
Yes	2 (1.2)	2 (0.5)	0.762
No	159 (98.8)	364 (99.5)	
Surgical history			
Yes	129 (80.1)	286 (78.1)	0.608
No	32 (19.9)	80 (21.9)	
Treatment history			
Chemotherapy			
Yes	33 (20.5)	85 (23.2)	0.521
No	128 (79.5)	281 (76.8)	
Radiotherapy			
Yes	9 (5.6)	23 (6.3)	0.759
No	152 (94.4)	343 (93.7)	
Chemotherapy and radiotherapy			
Yes	7 (4.3)	19 (5.2)	0.808
No	154 (95.7)	347 (94.8)	

## Discussion

In this prospective multicenter study, we evaluated the incidence rates and clinical manifestations of 5-FU- and capecitabine-associated cardiotoxicity in Chinese cancer patients. The highest incidence of symptomatic cardiotoxicity induced by 5-FU and capecitabine was 19.9% [11] and 34.6% [7] respectively in previous studies on other non-Chinese patient populations. In our study, the rates of 5-FU-associated cardiotoxicity was higher than that in previous reports (25.0% vs 19.9%) while cardiotoxicity associated with capecitabine occurred at a rate similar to previous studies (33.8% vs 34.6%). In our study, 4% of patients reported chest discomfort and palpitation whereas around 20% of patients presented arrhythmia. These rates were in a similar range as that reported previously [10]. Polk et al. reported that chest pain and palpitations were common symptoms during fluorouracil treatment with incidence rates from 0 to 18.6% and 0 to 23.1%, respectively, while arrhythmia was present in 0–21% of the patients [10]. Consistent with previous studies, ECG changes were not always associated with chest discomfort but they can reveal silent myocardial ischemia and asymptomatic arrhythmia in patients [12–14]. In our study, a substantial number of patients presented ischemic changes with no complaints of chest discomfort. We speculate that if patients on 5-FU or capecitabine treatment had been equipped with continuous ECG monitoring, the rate of cardiotoxicity observed could have been even higher given that many of ischemic episodes could be transient and silent [15]. Therefore, patients on 5-FU or capecitabine treatment should be closely monitored. In addition, we found a relatively high rate of abnormal ECG findings with around 13% of patients showing ST deviation compared with other studies [7, 16]. We reasoned that the discrepancy in clinical manifestation is partly due to our prospective study design.

5-FU and capecitabine can also cause serious cardiac events such as myocardial infarction and heart failure [4], with the latter also occurring more frequently in our study. In an exploratory step, we found that patients with pre-existing cardiac disease had a significantly higher risk of developing cardiotoxicity during 5-FU or capecitabine treatment, which was in line with previous reports [17, 18]. It should be noted that fluoropyrimidine-associated cardiotoxicity is potentially lethal even in patients without a prior history of cardiac disease. This is supported by the observation that around 5% of patients without prior cardiac disease developed heart failure and myocardial infarction on treatment with fluoropyrimidines. Capecitabine is an orally administered prodrug of 5-FU that is metabolized into the active form 5-FU in vivo through thymidine kinase conversion. Our study suggested that capecitabine is more prone to induce cardiotoxicity compared with 5-FU chemotherapy, consistent with the report of Kosmas et al. [8]. However, reports of other inconsistent results are also noted [9, 19]. We recommend careful monitoring of possible fluoropyrimidine-associated cardiotoxicity throughout the chemotherapy cycles and upon occurrence of cardiotoxicity, effective work-up should be applied, e.g. ECG monitoring, dose reduction of 5-FU or capecitabine, use of sublingual nitrates or calcium antagonists, and change to other chemotherapeutic drugs. Previous study suggested hypertension and chemotherapy cycles were potential risk factors of cardiotoxicity. We found that hypertension was significantly associated with cardiotoxicity in the univariate analysis. Although we failed to find such association in logistic regression model, this may be in part due to hypertension was in collinearity with other risk factors.

Fluoropyrimidine-associated cardiotoxicity has been indicated in a number of studies including ours, yet the pathogenesis is still unknown. Coronary vasospasm, endothelial injury and accumulation of toxic metabolites are the most common hypotheses for fluoropyrimidine-induced cardiotoxicity [20–22]. Guidelines for managing patients who develop cardiotoxicity following fluoropyrimidine treatment are still lacking. Collectively, there is a pressing need for alternative anticancer drugs that could substitute fluoropyrimidine agents for patients at high risk of intolerance towards them. Tegafur/gimeracil/oteracil (S-1) is a combination of three agents, comprising the oral fluoropyrimidine prodrug tegafur, the dehydropyrimidine dehydrogenase (DPD) inhibitor gimeracil (5-chloro-2,4-dihydroxypyridine) and potassium oxonate (OXO), an inhibitor of orotate phosphoribosyl transferase which converts 5-FU to fluorouridine monophosphate [23]. The clinical outcome of S-1 treatment is comparable with the results of 5-FU and capecitabine treatment. In particular, DPD inhibition by gimeracil is considered to significantly reduce F-beta-alanine and other cardiotoxic 5-FU catabolites like F-citrate, when comparing with capecitabine or 5-FU treatment, thus resulting in less cardiotoxicity [24]. However, DPD inhibition by gimeracil is partial and clinical evaluation of S-1-associated cardiotoxicity on a larger scale is still lacking. In a recent study, raltitrexed was found to show good safety in colorectal cancer patients [25]. Raltitrexed is an antifolate thymidylate synthase inhibitor that is also efficacious and safe in other tumor types including gastrointestinal tumor [26], head and neck cancer [27] and hepatocellular carcinoma [28]. Guidelines of both European Society for Medical Oncology (ESMO) and the National Institute for Health and Care Excellence (NICE) recommend that raltitrexed may serve as an alternative to 5-FU and capecitabine, both of which pose cardiotoxicity risk [29, 30]. In a phase III trial, first-line treatment with raltitrexed in combination with cisplatin demonstrated superior overall survival (OS) compared with cisplatin alone in the treatment of malignant pleural mesothelioma [31]. In addition, raltitrexed as first-line treatment of advanced colorectal cancer gave comparable OS as and a better safety profile than the 5-FU-based regimens in three different randomized clinical studies [32–34]. Gravalos et al. have also shown that raltitrexed/oxaliplatin (TOMOX) appears to have similar efficacy as FOLFOX4 and is well tolerated as the first-line treatment for advanced colorectal cancer. However, the two regimens offer different toxicity profiles, especially in grade 3 and 4 neutropenia and leukopenia. The convenient TOMOX regimen could be a useful alternative to fluoropyrimidine-based regimens [34]. Notably however, guidelines on dose reduction of raltitrexed should be strictly followed, especially in patients with impaired renal function, given that nearly half of the raltitrexed dose is excreted via the kidney in patients with normal renal function. The raltitrexed dose should be adjusted according to creatinine clearance with measurements conducted before the start of treatment and before each subsequent cycle [35].

There are several limitations in our study: S1 contains the fluoropyrimidine prodrug tegafur, which was not included in this study. Also, 5 patients (< 1%) were on bevacizumab combination therapy and we cannot exclude the possibility that bevacizumab may induce potential cardiovascular toxicity, with hypertension as the predominant symptom. Moreover, Hawthorne bias may occur in results potentially leading to a lower threshold for initiation of a cardiac work-up. In our study, however, cardiotoxicity was evaluated mainly based on objective indexes such as ECG and the doctor evaluated the overall condition of each patient and tried to reduce the risk of leading patients to a heightened awareness. We may thus have underestimated the incidence of cardiotoxicity in the patients, and further clinical evidence is required to confirm these results.

## Conclusions

Cardiotoxicity induced by fluoropyrimidine chemotherapeutic agents in the Chinese population may be underestimated in clinical practice. Close monitoring of patients is recommended, especially for those patients at high risk for cardiotoxicity.

## References

[1] Cardinale D, Colombo A, Lamantia G, Colombo N, Civelli M, De Giacomi G (2008). Cardio-oncology: a new medical issue. Ecancermedicalscience.

[2] Piedbois P, Rougier P, Buyse M, Pignon J, Ryan L, Hansen R, Zee B, Weinerman B, Pater J, Meta-analysis Group In C (1998). Efficacy of intravenous continuous infusion of fluorouracil compared with bolus administration in advanced colorectal cancer. J Clin Oncol.

[3] Hoff PM, Ansari R, Batist G, Cox J, Kocha W, Kuperminc M, Maroun J, Walde D, Weaver C, Harrison E (2001). Comparison of oral capecitabine versus intravenous fluorouracil plus leucovorin as first-line treatment in 605 patients with metastatic colorectal cancer: results of a randomized phase III study. J Clin Oncol.

[4] Saif MW, Shah MM, Shah AR (2009). Fluoropyrimidine-associated cardiotoxicity: revisited. Expert Opin Drug Saf.

[5] Wacker A, Lersch C, Scherpinski U, Reindl L, Seyfarth M (2003). High incidence of angina pectoris in patients treated with 5-fluorouracil. A planned surveillance study with 102 patients. Oncology.

[6] Tsavaris N, Kosmas C, Vadiaka M, Efremidis M, Zinelis A, Beldecos D (2002). Cardiotoxicity following different doses and schedules of 5-fluorouracil administration for malignancy—a survey of 427 patients. Med Sci Monit.

[7] Koca D, Salman T, Unek IT, Oztop I, Ellidokuz H, Eren M (2011). Clinical and electrocardiography changes in patients treated with capecitabine. Chemotherapy.

[8] Kosmas C, Kallistratos MS, Kopterides P, Syrios J, Skopelitis H, Mylonakis N (2008). Cardiotoxicity of fluoropyrimidines in different schedules of administration: a prospective study. J Cancer Res Clin Oncol.

[9] Jensen SA, Sorensen JB (2006). Risk factors and prevention of cardiotoxicity induced by 5-fluorouracil or capecitabine. Cancer Chemother Pharmacol.

[10] Polk A, Vaage-Nilsen M, Vistisen K, Nielsen DL (2013). Cardiotoxicity in cancer patients treated with 5-fluorouracil or capecitabine: a systematic review of incidence, manifestations and predisposing factors. Cancer Treat Rev.

[11] Khan MA, Masood N, Husain N, Ahmad B, Aziz T, Naeem A (2012). A retrospective study of cardiotoxicities induced by 5-fluouracil (5-FU) and 5-FU based chemotherapy regimens in Pakistani adult cancer patients at Shaukat Khanum Memorial Cancer Hospital & Research Center. J Pak Med Assoc.

[12] Rezkalla S, Kloner RA, Ensley J, Al-Sarraf M, Revels S, Olivenstein A (1989). Continuous ambulatory ECG monitoring during fluorouracil therapy: a prospective study. J Clin Oncol.

[13] Jeremic B, Jevremovic S, Djuric L, Mijatovic L (1990). Cardiotoxicity during chemotherapy treatment with 5-fluorouracil and cisplatin. J Chemother.

[14] Eskilsson J, Albertsson M, Mercke C (1988). Adverse cardiac effects during induction chemotherapy treatment with cis-platin and 5-fluorouracil. Radiother Oncol.

[15] Deedwania PC, Nelson JR (1990). Pathophysiology of silent myocardial ischemia during daily life. Hemodynamic evaluation by simultaneous electrocardiographic and blood pressure monitoring. Circulation.

[16] Akhtar SS, Salim KP, Bano ZA (1993). Symptomatic cardiotoxicity with high-dose 5-fluorouracil infusion: a prospective study. Oncology.

[17] Schober C, Papageorgiou E, Harstrick A, Bokemeyer C, Mugge A, Stahl M, Wilke H, Poliwoda H, Hiddemann W, Kohne-Wompner CH (1993). Cardiotoxicity of 5-fluorouracil in combination with folinic acid in patients with gastrointestinal cancer. Cancer.

[18] Labianca R, Beretta G, Clerici M, Fraschini P, Luporini G (1982). Cardiac toxicity of 5-fluorouracil: a study on 1083 patients. Tumori.

[19] Van Cutsem E, Hoff PM, Blum JL, Abt M, Osterwalder B (2002). Incidence of cardiotoxicity with the oral fluoropyrimidine capecitabine is typical of that reported with 5-fluorouracil. Ann Oncol.

[20] Mosseri M, Fingert HJ, Varticovski L, Chokshi S, Isner JM (1993). In vitro evidence that myocardial ischemia resulting from 5-fluorouracil chemotherapy is due to protein kinase C-mediated vasoconstriction of vascular smooth muscle. Cancer Res.

[21] Porta C, Moroni M, Ferrari S, Nastasi G (1998). Endothelin-1 and 5-fluorouracil-induced cardiotoxicity. Neoplasma.

[22] Sorrentino MF, Kim J, Foderaro AE, Truesdell AG (2012). 5-fluorouracil induced cardiotoxicity: review of the literature. Cardiol J.

[23] Deboever G, Hiltrop N, Cool M, Lambrecht G (2013). Alternative treatment options in colorectal cancer patients with 5-fluorouracil- or capecitabine-induced cardiotoxicity. Clin Colorectal Cancer.

[24] Yamada Y, Hamaguchi T, Goto M, Muro K, Matsumura Y, Shimada Y (2003). Plasma concentrations of 5-fluorouracil and F-beta-alanine following oral administration of S-1, a dihydropyrimidine dehydrogenase inhibitory fluoropyrimidine, as compared with protracted venous infusion of 5-fluorouracil. Br J Cancer.

[25] Ransom D, Wilson K, Fournier M, Simes RJ, Gebski V, Yip D (2014). Final results of Australasian Gastrointestinal Trials Group ARCTIC study: an audit of raltitrexed for patients with cardiac toxicity induced by fluoropyrimidines. Ann Oncol.

[26] Kelly C, Bhuva N, Harrison M, Buckley A, Saunders M (2013). Use of raltitrexed as an alternative to 5-fluorouracil and capecitabine in cancer patients with cardiac history. Eur J Cancer.

[27] Clarke SJ, Zalcberg J, Olver I, Mitchell PL, Rischin D, Dalley D (2000). Open label, multi-centre phase II study of raltitrexed (‘Tomudex’) in patients with inoperable squamous-cell carcinoma of head and neck. Ann Oncol.

[28] Zhao C, Fan L, Qi F, Ou S, Yu L, Yi X (2016). Raltitrexed plus oxaliplatin-based transarterial chemoembolization in patients with unresectable hepatocellular carcinoma. Anticancer Drugs.

[29] Schmoll HJ, Van Cutsem E, Stein A, Valentini V, Glimelius B, Haustermans K (2012). ESMO Consensus Guidelines for management of patients with colon and rectal cancer. a personalized approach to clinical decision making. Ann Oncol.

[30] National Institute for Health and Care Excellence (NICE): Managing advanced and metastatic colorectal cancer. 2017. https://pathways.nice.org.uk/pathways/colorectal-cancer/managing-advanced-and-metastatic-colorectal-cancer. Accessed 27 Mar 2018

[31] Van Meerbeeck JP, Gaafar R, Manegold C, Van Klaveren RJ, Van Marck EA, Vincent M, Legrand C, Bottomley A, Debruyne C, Giaccone G (2005). Randomized phase III study of cisplatin with or without raltitrexed in patients with malignant pleural mesothelioma: an intergroup study of the European Organisation for Research and Treatment of Cancer Lung Cancer Group and the National Cancer Institute of Canada. J Clin Oncol.

[32] Cunningham D, Zalcberg JR, Rath U, Oliver I, van Cutsem E, Svensson C (1996). Final results of a randomised trial comparing ‘Tomudex’ (raltitrexed) with 5-fluorouracil plus leucovorin in advanced colorectal cancer. “Tomudex” Colorectal Cancer Study Group. Ann Oncol.

[33] Cocconi G, Cunningham D, Van Cutsem E, Francois E, Gustavsson B, van Hazel G (1998). Open, randomized, multicenter trial of raltitrexed versus fluorouracil plus high-dose leucovorin in patients with advanced colorectal cancer. Tomudex Colorectal Cancer Study Group. J Clin Oncol.

[34] Gravalos C, Salut A, Garcia-Giron C, Garcia-Carbonero R, Leon AI, Sevilla I (2012). A randomized phase II study to compare oxaliplatin plus 5-fluorouracil and leucovorin (FOLFOX4) versus oxaliplatin plus raltitrexed (TOMOX) as first-line chemotherapy for advanced colorectal cancer. Clin Transl Oncol.

[35] Gunasekara NS, Faulds D (1998). Raltitrexed. A review of its pharmacological properties and clinical efficacy in the management of advanced colorectal cancer. Drugs.

